# The Role of Mitochondria in Carcinogenesis

**DOI:** 10.3390/ijms22105100

**Published:** 2021-05-12

**Authors:** Paulina Kozakiewicz, Ludmiła Grzybowska-Szatkowska, Marzanna Ciesielka, Jolanta Rzymowska

**Affiliations:** 1 Department of Radiotherapy, Medical University in Lublin, Chodźki 7, 20-093 Lublin, Poland; ludmila.grzybowska-szatkowska@umlub.pl (L.G.-S.); marzanna.ciesielka@umlub.pl (M.C.); 2 Department of Radiotherapy, St. John’s Cancer Centre, The Regional Oncology Centre of Lublin Jaczewskiego 7, 20-090 Lublin, Poland; 3 Chair and Department of Forensic Medicine, Medical University in Lublin, Jaczewskiego 8b, 20-090 Lublin, Poland; 4 Chair and Department of Biology and Genetics, Medical University of Lublin, Chodźki 4A, 20-093 Lublin, Poland; jolanta.rzynowska@umlub.pl

**Keywords:** mtDNA, polymorphism, cancer, mutagenesis

## Abstract

The mitochondria are essential for normal cell functioning. Changes in mitochondrial DNA (mtDNA) may affect the occurrence of some chronic diseases and cancer. This process is complex and not entirely understood. The assignment to a particular mitochondrial haplogroup may be a factor that either contributes to cancer development or reduces its likelihood. Mutations in mtDNA occurring via an increase in reactive oxygen species may favour the occurrence of further changes both in mitochondrial and nuclear DNA. Mitochondrial DNA mutations in postmitotic cells are not inherited, but may play a role both in initiation and progression of cancer. One of the first discovered polymorphisms associated with cancer was in the gene NADH-ubiquinone oxidoreductase chain 3 (mt-ND3) and it was typical of haplogroup N. In prostate cancer, these mutations and polymorphisms involve a gene encoding subunit I of respiratory complex IV cytochrome c oxidase subunit 1 gene (*COI*). At present, a growing number of studies also address the impact of mtDNA polymorphisms on prognosis in cancer patients. Some of the mitochondrial DNA polymorphisms occur in both chronic disease and cancer, for instance polymorphism G5913A characteristic of prostate cancer and hypertension.

## 1. Introduction

Cancer is a group of diseases characterised by uncontrolled abnormal cell growth. In 90–95%, cancer is caused by genetic mutations associated with environmental and lifestyle factors, while 5–10% is connected with inherited genetics [1]. The activation of oncogenes and the inactivation of tumour suppressor genes contributes to carcinogenesis. Various mutations are reported in well-characterised cancer genes but the whole process of carcinogenesis remains a mystery [2]. The genome instability leads to cancer development by tumour angiogenesis, uncontrolled proliferation of cancer cells which are resistant to death and avoid growth suppressors. Malignant cells can invade nearby tissue or metastasize and avoid the immune system [3]. Furthermore, carcinogenesis leads to changes in energy metabolism of cells [4]. Growing tumour cells produce a high level of lactate during aerobic glycolysis. Tumour mitochondria are characterised by an increased level of ROS, hypoxia and signals inhibiting apoptosis [5]. Most of the mtDNA somatic mutations in tumours have been also reported as polymorphisms in the general population. MtDNA mutations occur in various diseases. They are a disorder associated especially with metabolism and energy [6]. In this review, we focus on the changes in mitochondrial DNA associated with cancer (and other diseases), as well as on the function of mitochondria in human cells.

## 2. Mitochondrion as Organelle

The mitochondrion is an organelle that, via cell oxidation processes, is a key energy converter in eukaryotic cells. The mitochondria synthesise adenosine triphosphate (ATP), an energy carrier used in various cellular processes, via the mitochondrial electron transport chain in the process of oxidative phosphorylation (OXPHOS) [7,8]. The respiratory chain consists of four protein complexes located in the mitochondrial inner membrane. Electrons are transported onto an oxygen molecule with the participation of the first four complexes. Complex I is called reduced nicotinamide adenine dinucleotide (NADH)-ubiquinone oxidoreductase (Q reductase), complex II is known as succinate-ubiquinone oxidoreductase (SQR) and the third is ubiquinol-cytochrome c reductase complex, while the fourth is called cytochrome c oxidase. Transport of reducing equivalents leads to proton gradient formation enabling ATP synthesis via activation of ATP synthase also known as complex V [9,10]. The mitochondria are also engaged in nucleotide, amino acid and lipid metabolic pathways. Moreover, beta oxidation of fatty acids, the Krebs cycle, takes place in the mitochondrial matrix [10]. The mitochondria participate in maintaining the cell calcium balance and regulate the cell’s redox potential by taking part in reactive oxygen species formation [11,12]. When discussing the role of the mitochondria in carcinogenesis, it must be noted that they participate in the intrinsic apoptotic pathway [13].

## 3. Mitochondrial DNA

The mitochondrion is a semi-autonomous organelle with its own genome. It is not, however, completely independent of nuclear DNA (nDNA) [14]. Mitochondrial DNA (mtDNA) is a circular, supercoiled molecule consisting of 16,569 base pairs and located in the mitochondrial matrix [15,16]. It contains 37 genes without introns. They encode 13 polypeptides that create protein respiratory chain complexes, two rRNA molecules (12S rRNA, 16S rRNA) and 22 tRNA molecules [17]. The remaining proteins are coded by nDNA [14]. Mitochondrial DNA contains several ultra-short intergenic non-coding regions and one large region in the form of a D-loop consisting of 1122 base pairs. The D-loop encompasses nucleotides 16,024–16,569 and 1–576, takes part in mitochondrial genome replication and contains sequences that are promoters of genes coded by mtDNA. Two hypervariable regions (HVR) can be distinguished in its structure, HVRI and HVRII [17].

The first complete sequence of the human mitochondrial genome was published in 1981 by Anderson et al. [18]. Currently, the reference mtDNA sequence is the one updated in 1999, called the Revised Cambridge Reference Sequence rCRS, which is available in the GenBank database under the accession number NC_012920 [17,18].

## 4. Mitochondrial Haplogroups and Polymorphisms

In humans, mitochondrial DNA is not inherited in accordance with the Mendel’s principles, but passed on to the next generation only in the maternal lineage without recombination. This hereditary difference and the frequency of mutations make mtDNA analysis a valuable tool in studies on the origins of contemporary people. The human mtDNA classification system consists of a network of haplogroups marked with capital Roman letters [19]. Haplogroups are defined as groups of haplotypes with the same several polymorphisms. Characteristic mitochondrial mutations, which occurred in a given place and at a given time, were accumulated through maternal lineages during the migration of the human population and have led to the formation of haplogroups [6]. This is the basis for the assessment of geographical distribution of populations and human migration [19,20]. Genetic polymorphism occurs by way of an alteration in the DNA that is usually a result of cell adaptation to changing environmental conditions. The variation of genotypes is inherited from parents and occurs in all body cells. A polymorphism is present when the observed variability between individuals is not maintained by a recurrent mutation [21]. The most frequent variability seen in the human genome is single nucleotide polymorphism SNP [21,22]. The oldest mitochondrial group that gave rise to the next ones is haplogroup L0. It is typical for the Bushmen of South Africa and the Sandawe people of East Africa [23,24]. Seven main mitochondrial groups have been distinguished based on mtDNA: L0, L1, L2, L3, L4, L5, and L6. Haplogroup L3 gave rise to all mtDNA macro-haplogroups beyond Africa and is divided into two subclades, M and N. Group N is an ancestor of haplogroups R, X, I, W, A, S, O, and Y, while clade M produced haplogroups Q, E, G, D, C, and Z [23,24]. Haplogroup R initiated nine other haplogroups H, U, K, J, T, V, F, B, and P. In Europe, the prevailing haplogroups are those descended from the macro-haplogroup N, H, U, K, J, T, V, X, I, and W [20,25]. In Asia, however, the predominant haplogroups are A, S, O, and Y, which originate from the macro-haplogroup M [25]. Within haplogroups, there are haplotypes, i.e., sets of polymorphisms that distinguish an individual from rCRS. The mtDNA rCRS sequence belongs to haplogroup H, which is the most common haplogroup in Europe [20,26].

## 5. Heteroplasmy and Homoplasmy of mtDNA

Mitochondrial DNA is characterised by significantly greater occurrence of mutations than nDNA [16,20]. This considerable susceptibility to chemical and physical factors is assumed to be associated with the lack of protective histones in its structure and, hence, increased exposure to reactive oxygen species (ROS) that form in the respiratory chain [26,27,28]. However, histones are also reported to be capable of promoting DNA damage under the influence of certain conditions and factors [29,30]. MtDNA binds to a protein to form a complex known as a mitochondrial nucleoid. These proteins can protect and stabilize mtDNA [30].

A eukaryotic cell contains usually identical copies of mtDNA, hence the term mtDNA homoplasmy [26,31,32]. External factors may lead to mutations and formation of altered mtDNA. Such a cell contains both mutated and wild-type mtDNA. This situation is referred to as heteroplasmy. During mitotic division, mutated mtDNA is inherited by daughter cells in humans. This may result in the preponderance of mutated mtDNA over wild-type mtDNA, and entail a change in the cell’s phenotype. To enable phenotypic expression of altered mtDNA in an adult individual, the number of mutated copies must be greater than that of wild-type copies [31,32,33]. The intensity and type of clinical symptoms can depend on the degree of heteroplasmy and change with the patient’s age [31,32]. Mitochondrial DNA mutations have been described in various disease entities with underlying dysfunction of the respiratory chain [29,31]. The pathology of these diseases is associated with impaired functioning of organs made of tissues of high energy requirement, containing the greatest number of mitochondria. These mostly include the brain, skeletal muscles and eyes [29,31]. It should be noted that mitochondrial diseases are characterised by genetic heterogeneity and pleiotropy. A single mutation in one gene manifests itself with numerous clinical effects for various tissues. The mutant gene causes many symptoms independent of one another. An example can be the A3243G mutation in the MT-TL1 gene, which manifests itself in various diseases–MELAS, diabetes with deafness, mitochondrial myopathy, gastrointestinal or renal symptoms even in the same family [34]. Another problem is the relationship with gender and morbidity of certain mitochondrial diseases. An example of incomplete penetration of mutated genes of mitochondrial DNA associated with sex can be found in Leber’s hereditary optic neuropathy (LHON). About 50% of men and about 10% of women with a pathological change in mtDNA will experience optic neuropathy [35]. This suggests that there must be additional mitochondrial and nuclear, and perhaps even environmental factors modifying the phenotype of LHON disease [36].

## 6. Mitochondrial DNA and Carcinogenesis

### 6.1. Mitochondrion Metabolism and Cancer

**Figure 1:**
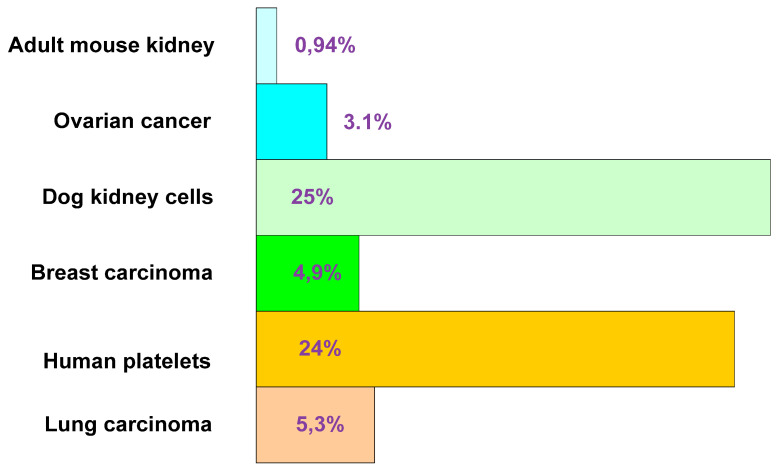
Glycolytic ATP contribution in tumour and normal cells expressed as a percentage of total ATP production (based on Zu and Guppy [38]).

**Figure 2:**
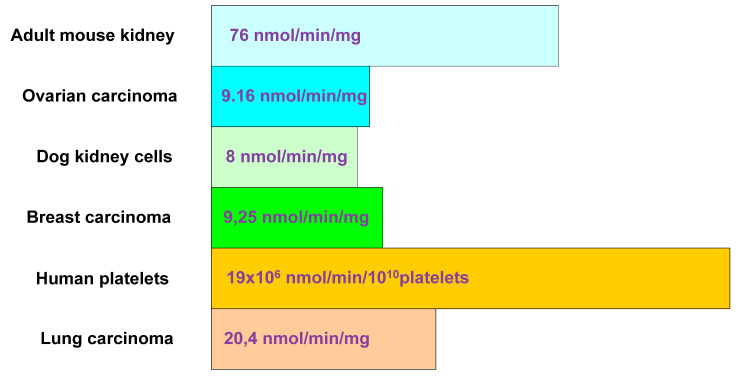
Glycolytic ATP contribution in tumour and normal cells–Oxidative ATP production (based on Zu and Guppy [38]).

An interest in the mitochondria in the context of carcinogenesis was initiated at the beginning of the 20th century by Otto Warburg, who noticed the predominance of the glycolysis process in cancer cells despite the availability of oxygen (aerobic glycolysis) [4]. The atypical physiology of cancer cells prompted the German biochemist to put forward a hypothesis about a significant role of cell respiratory disorders in carcinogenesis [4,37]. However, the mechanism of this aberrant metabolic state remained a mystery. The contribution of glycolysis to ATP production appears to depend on the type of tissue (Figure 1 and Figure 2). The mean values of glycolytic distribution in total ATP production are 20% for normal cells and 17% for cancer cells [38].

**Figure 3:**
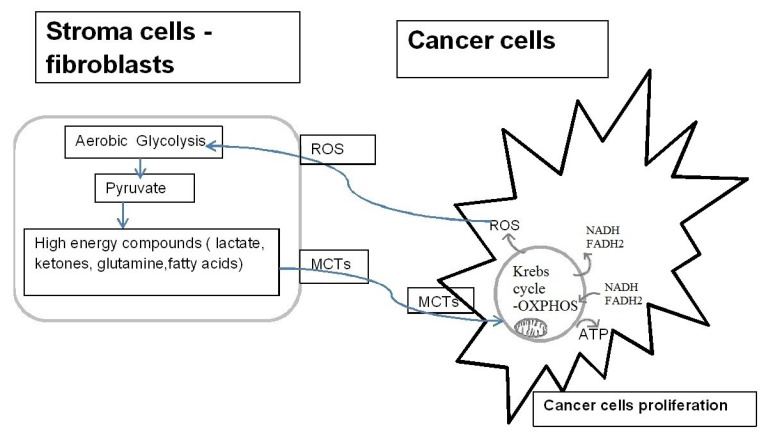
Reverse Warburg effect. Reactive Oxygen Species (ROS) secreted by cancer cells induce aerobic glycolysis in neighboring stromal fibroblasts. The high-energy compounds produced as a result of aerobic glycolysis are transported back to the cancer cells. These compounds are to be used for anabolic processes and the formation of ATP in the process of aerobic respiration by cancer cells, which is assumed to contribute to the progression of cancer. MCTs-monocarboxylate transporters,-plasma membrane transporters that carry molecules having one carboxylate group.

It is now believed that the Warburg effect occurs only in fibroblasts in the tumour cell stroma. Aerobic glycolysis is thought to occur in fibroblasts that provide cancer cells with high energy compounds such as lactate, ketones and glutamine [38]. These compounds are presumably used for anabolic processes and the formation of ATP in the process of aerobic respiration by cancer cells, which is assumed to contribute to the progression of cancer. This process is called the reverse Warburg effect (Figure 3). In addition, the Crabtree effect can occur in cancer cells. Cancerous and rapidly proliferating cells can reversibly convert the fermentation process into oxidative metabolism, depending on the presence of glucose in the cell or in its environment [39,40].

In the presence of glucose, after a short period of activation of oxidative phosphorylation, the energetic metabolism switches from oxygen to glycolytic and inhibits the intensity of cellular respiration. It is accompanied by acidification of the environment and reduction of nicotinamide nucleotides. The possibility of reversible suppression of oxidative phosphorylation can be an advantage of tumour cells in vivo due to the adaptation of their metabolism to inhomogeneous conditions of the microenvironment of malignant tumours [39,40]. The level of phosphate and calcium ions can also influence the Crabtree effect in a cancer cell. In tumour cells, after the addition of glucose, the level of phosphate ions decreases and, as a result, the thermodynamic phosphate potential changes [40]. The increase in glucose levels may also be accompanied by Ca^2+^ accumulation. The Ca^2+^ influx inhibits oxidative phosphorylation by inhibiting ATP synthase in tumour cells (as in the mitochondria of normal tissues) [41,42]. Due to the activation of glycolysis, the supply of ADP adenosine diphosphate and phosphate, whose deficiency should limit oxidative phosphorylation, is reduced. Oxidative phosphorylation can be regulated by the mitochondrial outer membrane that regulates substrate access to the intermembrane space [40].

Hexokinase II binds to the external mitochondrial membrane at the fusion sites between the outer and inner membranes. Based on studies on hepatocellular cancer cell metabolism, it was found that the predominance of glycolysis is associated with a disturbed ratio of expressed enzymes, more precisely hexokinases [43,44]. By contrast with healthy cells, cancer cells present a predominance of hexokinase II over hexokinase IV. This is undoubtedly associated with the fact that mutated TP53 or hypoxia typical of cancer cells have a positive effect on hexokinase II expression [43,44]. When hexokinase II undergoes phosphorylation, it develops a capacity to directly bind with an ion channel of the mitochondrial membrane that participates in ATP-dependent transport (voltage-dependent anion channel, VDAC) [43]. As a result, glucose is rapidly phosphorylated to glucose 6-phosphate, and hexokinase II loses its sensitivity to inhibition by G-6-phosphate. In this mechanism, cancer cell metabolism changes from aerobic to anaerobic. Additional significant consequences that should be mentioned here are the blockage of the Bax and Bak proteins, which take part in apoptosis induction, and stabilisation of mitochondrial permeability transition pores (mtPTP) [37,43,44].

### 6.2. Changes in Mitochondrial DNA and Carcinogenesis

Mutations in mtDNA that occur in germ cells or are inherited in the maternal lineage may increase predisposition to a given cancer. Moreover, mtDNA mutations in postmitotic cells are not inherited, but may play a role both in initiation and progression of cancer [45,46]. A role of the mitochondrial genome in carcinogenesis is also supported by a relationship between the presence of a circular dimer form–a double-length circular molecule–and complex catenated forms of mitochondrial DNA in leukemic leukocytes and a severe course of granulocytic leukaemia [47]. It seems that these mutations are of de novo nature as they appear in the form of slowly progressing clinical symptoms, probably a long time after the occurrence of mutation (when mutated DNA becomes prevalent over wild-type DNA) [45,46]. As most cancerous mutations are homoplasmic, there must be an adequate number of cell divisions, and altered mtDNA must be sent to most mitochondria [48,49]. The conventional homoplasmy model assumes the occurrence of intramitochondrial segregation and predominance of one type of mtDNA in the mitochondria, so-called functional advantage [48,49]. At the cellular level, the mitochondria with one type of mtDNA become prevalent. During cell division, replicative segregation takes place. It consists in a change of proportions between mutated and normal mtDNA, with the predominance of altered mitochondria (selective advantage). This leads to the presence of only one type of mtDNA in subsequent cell divisions, i.e., homoplasmy [46,48,49]. In the mathematical model, cell homoplasmy is an effect of incidental mitochondrial segregation that occurs during subsequent divisions (genetic drift) [50,51]. As a result of the genetic drift, rare mtDNA variants can be either eliminated or fixed. Selective growth of cells with mutated mtDNA may also result from changes in nDNA [46,48]. Nuclear DNA may stimulate proliferation of cells with altered mitochondria, yielding daughter cells with one type of mtDNA, which initiates tumour growth [45,50]. The period needed to reach the predominance of mutated mtDNA or cell homoplasmy would correspond to the phase of cancer transformation [46,48]. The expression of changes in mtDNA also depends on the type of gene affected, type of mutation, type of tissue in which mutation occurs and its energy requirement, as well as the mitochondrial subgroup to which a given individual belongs. Moreover, the coexistence of mtDNA polymorphisms or their specific configuration may also be significant in cell dysfunction [45,46]. Mutations occurring in mtDNA can be beneficial, neutral or harmful [46,49]. Humans adjust to the changing environment thanks to mtDNA mutations. During evolution, mutations enabled adaptation to the changing climate. However, they can also become a cause of predisposition to certain diseases, including cancers [45,48]. As for carcinogenesis, two types of mtDNA mutations are distinguished, tumorigenic and adaptive. The harmful tumorigenic group includes end-chain mutations and missense mutations, which lead to changes in a coded amino acid in a protein, and point deletions or insertions. The effects of these mitochondrial genetic changes may lead to impaired functioning of the respiratory chain, entailing an increase in reactive oxygen species, which may contribute to further mtDNA mutations. The developing free radicals may be both initiators and promoters of carcinogenesis [29,37]. Both in nDNA and in mtDNA, guanine residues are preferentially damaged by free radicals, which induces G > T transversions [28,29,49]. However, age-related point mutations of mtDNAs are characterised by mainly G > A transition and only a small percentage of G > T transversion [52,53,54], which may be caused by transcription errors. In the case of colorectal cancer, 70% of mutations involved T to C and G to A replacement [51]. Adaptive mutations result from cell adaptation to changing environmental conditions. These include missense mutations or control region mutations that lead to replication and transcription modulation, making a cancer cell adjust to unfavourable bioenergetic conditions during metastasising [45,47].

In the studies on the role of mtDNA in carcinogenesis, the Hayashi group demonstrated mutagenicity of the mutations G13997A and 13885insC in the *ND6* gene [55,56]. Both of these mutations were responsible for deficiency in respiratory complex I and increased production of ROS in the high-metastatic line carcinoma A11 (Lewis lung carcinoma and high-metastatic line fibrosarcoma P82M) [57]. Cell lines (i.e., low-metastatic line carcinoma P29) without these mutations did not show overproduction of ROS [56]. Application of ROS scavenger in the P29mtA11 cybrids reduced both the ROS production and the metastatic potential in mouse model [57,58]. Similarly, mtDNA ATP6 mutations (T8993G) into the PC3 prostate cancer cell line result in both an increase in ROS production and faster tumour growth, compared to wild-type hybrids in mice [59].

Furthermore, changes in mtDNA copy number in cells are reported in cancer. There is some conflicting information about the number of mtDNA copies in cancer cells [60,61]. For example, an increase was reported in papillary thyroid carcinoma and primary head and neck squamous cell carcinoma, while in gastric cancer a depletion was observed [61,62]. The number of mtDNA copies in cancer cells may be associated with a specific site of mutation related to the type of tumour [61]. Mutations in nuclear genes or response to mtDNA impairment may have a role in controlling mtDNA copy number. However, the number of copies may affect the expression of respiratory genes, exogenous and environmental influences should also be taken into account [6]. In a study on three models of tumourigenesis (i.e., glioblastoma multiforme, multiple myeloma and osteosarcoma), it was shown that mtDNA copy number has an influence on early and late cancer progression [63]. Tumour cells with a decreased number of mtDNA copies restored their number, which increased the tumour growth rate [61]. MtDNA copy number in tumour cells can have an influence on the onset of cancer [6]. For example, a decreased mtDNA copy number in colorectal cancer was associated with lymph-node metastasis and lower survival rates [64]. In another study, it was shown that mtDNA depletion may have an important role in tumour progression because of a correlation with aberrant nuclear-encoded genes [65]. The mechanism of changes in the number of mtDNA copies in cancer cells is not yet well understood [66].

Additionally, when describing mitochondrial DNA variants, we should mention the mitochondrial-nuclear crosstalk phenomenon which is important for the maintenance of cellular homeostasis. Mitochondrial dysfunction, caused by mtDNA modification, can contribute to epigenetic changes in the nuclear genome, such as DNA and chromatin alterations and singling through small RNA. This, in turn, may lead to the continuance of the oncogenic transformation initiated by the mitochondrion. The process of signal transmission is not fully understood [66].

### 6.3. mtDNA Polymorphisms and Haplogroups Associated with Cancer

One of the first discovered polymorphisms associated with cancer was characterised by a replacement of guanine with adenine at position 10,398, leading to a change in codon A114T (A–alanine, T–threonine) in the ND3 gene–G10398A. It caused alterations in the respiratory complex I [67]. This polymorphism presumably increases the likelihood of invasive breast carcinoma in African American women, compared with women with guanine at this position (G10398A). This relationship, however, does not supposedly concern the Caucasian race [67]. Based on a study involving a Polish population, the A10398G polymorphism is associated with greater incidence of this cancer [68]. The same conclusion was drawn from a study evaluating a non-Jewish European–American population [69]. Additionally, it showed that a higher risk of breast cancer was associated with mitochondrial haplogroup K, while the lowest risk was noted in haplogroup U [69]. Apart from the A10398G polymorphism, two other polymorphisms have been associated with an increased risk of breast cancer, T16519C and G9055A [69]. Polymorphisms T3197C (OR = 0.31, 95% CI, 0.13–0.75, *p* = 0.0043) and G13708A (OR = 0.47, 95% CI, 0.24–0.92, *p* = 0.022) supposedly lower this risk [69]. In one of the studies conducted in a Polish population, involving mutations and polymorphisms within the MT-ND1, MT-ND-2, MT-ND3, and MT-ND6 genes in breast cancer cells, there were 28 polymorphisms, mostly located within the MT-ND-2 gene, that were absent from healthy cells [70]. Some of them are associated with mitochondrial diseases, such as C4640T, often occurring in patients with LHON [71]. The G10398A polymorphism increases the risk of non-small cell lung cancer (NSCLC) in haplogroup N and oral cancer in haplogroup M [72,73]. It must be underlined, however, that there are studies that do not corroborate the role of polymorphisms, A10398G in particular, in breast cancer [74,75]. As for prostate cancer, studies investigating mutations and polymorphisms within a gene encoding subunit I of respiratory complex IV *COI* are significant [76,77].

Four non-synonymous mutations in the COI gene, associated with given mitochondrial haplogroups, have been discovered in prostate cancer cells [59]. T6253C mutation was detected in cancer cells of haplogroup H individuals. C6340T mutation was found in haplogroups J, T, L1 and N, G6261A mutation occurred in haplogroups H and N, while A6663G mutation was discovered in patients with haplogroups L0 and L2 [59]. These are homoplasmic changes, which attests to positive selection of mutated mtDNA in subsequent cell divisions [68,78]. When comparing the incidence of COI polymorphisms in the African and American population of European ancestry, the rates were 17.4% and 6.5%, respectively, suggesting a higher risk of prostate cancer in the former group [79]. Polymorphisms T6221C and T7389C are significantly associated with prostate cancer (*p* < 0.05) [79]. Detected mutations, such as G5949A (G16X), T6124C (M74), C6924T (A341S), also concern highly conserved amino acids examined in an American population (482 men with prostate cancer and 189 men without cancer), where COI polymorphisms were detected in 8.8% of Caucasian patients with prostate cancer and in 72.8% of cancer patients of African ancestry [80]. Moreover, the rate of polymorphisms in the Caucasian controls was 0.0% compared with 64.3% in African Americans [80]. Missense mutations in the COI gene occurred in 116 of 482 (24.2%) patients with prostate cancer [80]. Detected A5935G and G5949A mutations were related to highly conserved amino acids (CI–conservation index 100%) [80]. In a study conducted among white inhabitants of North America, mitochondrial haplogroup U was linked with an increased risk of prostate and renal cancers [81]. However, subsequent studies did not support the relationship of prostate cancer with haplogroup U [81].

In a study involving a South European population with vesicular and papillary thyroid carcinoma, the authors reported a protective role of mitochondrial haplogroup K in relation to these cancers [82]. Furthermore, Chinese reports indicate a relationship between haplogroup D4a and increased incidence of thyroid carcinoma [59]. This study also showed an increased risk of breast cancer in haplogroup M, particularly when women also belonged to subgroup D5 [83]. Haplogroups M7 and M8 increased the risk of hepatocellular carcinoma [84].

The D-loop is highly polymorphous, and the literature reports the presence of numerous changes in this region in various cancers [85,86]. These changes have been reported for ovarian carcinoma [85], oesophageal cancer [86], glioma [78], renal cancer [87], hepatocellular carcinoma [88] and others. Polymorphisms in the D-loop region such as 73G/A, 146T/C, 195T/C, 324C/G, 16261C/T, and 16304T/C are associated with an increased risk of developing colon cancer [89].

One of the studies on a Polish population concluded that haplogroup H with the C7028T polymorphism may be considered a protective factor in endometrial cancer [90]. This polymorphism has also been reported in patients with sensorineural hearing loss [91,92]. The C7028T polymorphism is diagnostic for mitochondrial haplogroup R and referential for subgroup H [92]. Czarnecka et al. [90] also found an association between the occurrence of three polymorphisms, 16223C, 16126C and 207A, in the D-loop region of mtDNA in endometrial cancer. A polymorphism consisting in T > C transition in the highly polymorphous D-loop region 16,189 is linked not only with type 2 diabetes mellitus, but also with increased incidence of endometrial cancer, glioma and breast cancer [93,94,95]. One of the studies addressing breast cancer and mtDNA mutations within the D-loop and MT-ND4 gene revealed correlations between BRCA1 mutation and haplogroup X, and between BRCA2 mutation and haplogroup H [96]. 

The occurrence of single nucleotide polymorphisms within the D-loop is said to increase the risk of renal-cell carcinoma [87]. Alterations in nucleotide sequences 16,293 A > G and 262 A > G concern clear-cell renal carcinoma, while 488 T > C refers to papillary tumours and benign oncocytoma [87]. As for urinary bladder cancer, there are reports about its relationship with the D-loop C16069T polymorphism [97]. Haplogroup M7b2 presumably increases the risk of haematological cancers [97]. The A12308G polymorphism, which is a marker of haplogroup U and relates to the tRNA gene for leucine 2, is said to raise a risk of renal and prostate cancers (odds ratio OR = 2.52 and 1.95, respectively) [97].

In research on mitochondrial polymorphisms in cancer, some authors went even further and searched for their influence on prognosis. In gastric carcinoma, survival was longer in patients with haplogroup N (489T) than in patients with haplogroup M [98]. As for hepatocellular carcinoma, patients with cytosine at position 146 in mtDNA were characterised by shorter survival, compared with patients with thymine at this position (relative risk, 2.781, 95% CI, 1.127–6.859, *p* = 0.026) [98]. Three other mutations (T15784C, C16185T, A16399G) were associated with a better prognosis in patients with haplogroup M7 and M8 [84]. In colon cancer and nSCLC, mutations detected in ND genes correlated with the presence of distant metastases (*p* < 0.05) [99].

When investigating a relationship between single nucleotide polymorphisms and survival in small-cell lung carcinoma, one study among Chinese people revealed longer survival in patients with the 16390A polymorphism, compared to those with guanine at this position [100]. In the case of colorectal carcinoma, the 10398 A > G polymorphism, leading to the replacement of the amino acid in subunit ND3 (T114A), is said to be conducive to distant metastases [101]. This polymorphism has also been discovered in women with breast cancer [67,68]. However, there was no relationship of overall survival and progression-free survival in colorectal carcinoma with this polymorphism and 5 other polymorphisms, T479C, T491C, T10035C, A13781G, and T16189C [101]. As for prostate cancer, prognosis in patients with haplogroup L0, and particularly L0d, was worse than in the case of patients with haplogroups other than L0. These patients had higher Gleason Score (6.3 versus 4.9, *p =* 0.049) [77]. A summary of changes in mtDNA associated with cancer is presented in Table 1.

## 7. Mitochondrial Haplogroups and Chronic Diseases

Individual mitochondrial haplogroups can be associated with a risk of developing certain chronic diseases. The occurrence of a polymorphism in the HVRI region of mtDNA in the form of T > C transition at position 16,189 may increase the risk of type 2 diabetes mellitus, which has been shown in a study involving a European population [94,111]. This has also been confirmed in an Asian group, where it was additionally found that haplotype B4 with SNP T16189C is associated with greater susceptibility to type 2 diabetes mellitus [111,114]. This polymorphism has also been linked with an increased incidence of MELAS syndrome [115]. Neurodegenerative conditions, in which the mitochondria undoubtedly play a significant pathogenetic role, are being investigated by scientists studying mtDNA. It has been noted that the risk of Parkinson’s disease is higher in people with haplogroup J and T characterised by T to C substitution at position 4216 of the *ND1* gene in mtDNA, which results in a replacement of tyrosine to histidine at position 304 of the protein and leads to reduced activity of this complex in affected cells, thereby increasing the frequency of ND1 mutation [116,117]. This polymorphism is also said to be associated with LHON and insulin resistance [115,116]. Individuals with haplogroup J in the European population have also been observed to carry an increased risk of LHON syndrome, which is associated with more frequent 14484T > C mutation in the mitochondrial *MT-ND6* gene [118]. However, other authors argue that the association of LHON with any haplogroup is weak, which supports the need for more research on mtDNA polymorphisms [119]. A study in a Portuguese population revealed a lower risk of ischaemic stroke in haplogroup H1 [120]. Subhaplogroup K, in turn, has been considered a risk factor of ischaemic heart disease and transient ischaemic attacks (TIA), but not of acute coronary syndrome [121]. As for neurodegenerative conditions, one of the studies involving a European population has shown an increase in the risk of Alzheimer’s disease among males from haplogroup U, compared to haplogroup H, the most common haplogroup in Europe [122]. As already mentioned, diseases caused by mutations in the mitochondrial DNA in most cases give very different symptoms, even in members of the same family. Environmental factors can also affect the occurrence of the symptoms of a specific mutation. A strong correlation was found between cigarette smoking and LHON symptoms [112,113]. Smoking increases the risk of blindness [123,124]. It is possible that metabolic processes occurring in cells contribute to the modification of accumulating mutations in mitochondrial DNA, which translates into the phenotype of patients. It cannot be ruled out that coexistence of additional sense-type mutations may be responsible for the way these mutations occur.

All the factors described above, such as gender, penetration, pleiotropy, tissue type, type of mutation, may be responsible for the way the mtDNA mutation is revealed. It may also explain why the increased risk of cancer has not been reported in a group of patients with mitochondrial disease yet. The understanding of these mitochondrial DNA dysfunctions is still incomplete.

## 8. Conclusions

Without a doubt, the mitochondria are essential for normal cell functioning, including its apoptosis. That is why changes in mitochondrial DNA can cause chronic diseases. The role of mtDNA changes in carcinogenesis is complex and not entirely understood. Belonging to a given mitochondrial haplogroup may, on the one hand, be conducive to cancer development, but on the other hand, it may reduce the risk of its occurrence. A predisposition to cancer may be due to the inheritance of certain mother-child polymorphisms, such as in mitochondrial disease. Mutations in mtDNA occurring via an increase in reactive oxygen species may be a factor contributing to further changes both in mitochondrial and nuclear DNA. It cannot be excluded that cells with altered respiratory chain proteins may also prevail in the selection process in the conditions of lower oxygen supply in a tumour. Further mtDNA analyses in the context of carcinogenesis should be conducted due to the potential of using mtDNA alterations in molecular diagnosis of cancers and in order to provide a profound explanation of the very process of carcinogenesis. Mitochondrial DNA polymorphisms may be used as tumour markers and as prognostic factors in patients with malignant diseases.

## Figures and Tables

**Table 1:** The resume of changes in mtDNA associated with cancer.

Polymorphism	Mitochondrial Gene	Type of Mutation	Amino Acid Change	Haplogroup	Type of Cancer	Chronic Disease
T6253C	MT-CO1	missense	M ^117^ T	H, L1, M, D, A	prostate cancer [59]	-
C6340T	MT-CO1	missenese	T ^146^ I	J, T, L1, N, E,	prostate cancer [59]	-
G6261A	MT-CO1	missenese	A ^120^ T	N, L3, C, R, H, J, T, B	prostate cancer [59]	LHON [102]
A6663G	MT-CO1	missenese	I ^254^ V	L2	prostate cancer [59]	-
C5911T	MT-CO1	missenese	A ^3^ V	L0, R, H	prostate cancer [59]	-
A7158G	MT-CO1	missenese	I ^419^ V	L3, N, R	prostate cancer [59]	-
A6047G	MT-CO1	synonymic	L ^48^ L	U	pancreatic cancer [49]	-
T5999C	MT-CO1	synonymic	A ^32^ A	U, M, H	pancreatic cancer [49]	-
G5913A	MT-CO1	missenese	D ^177^ N	F, K	prostate cancer [59]	hypertenssion) [103]
G9055A	MT-ATP-6	missenese	A ^177^ T	M, Z, A, R, H, J, B, U	breast cancer	PD protective factor [104]
A10398G	MT-ND3	missenese	T ^114^ A	N, S, N, W, Y, X, R, J, B, K, U	breast cancer [69]	LHON, PD protective factor, ADHD [105], metabolic syndrome [106]
G10398A	MT-ND3	missenese	A ^114^ T	N	breast cancer, esophageal cancer [107], non-small cell cancer [108]	-
G14905A	MT-CYB	synonymic	M ^53^ M	B, T, D, L0, L2, L3, L4, L5	breast cancer [69]	-
C14766T	MT-CYB	missenese	I ^7^ T	-	breast cancer [69]	-
C16270T	D-loop	synonymic	not applicable	L1, L3, M, D, N, I, A, P, H, T, U, K	melanoma [109]	-
A16183C	D-loop	synonymic	not applicable	-	melanoma [109]	-
C16192T	D-loop	synonymic	not applicable	L0, L2, L3, M, C, S, I, W, A, X, P, V, H, J, T, R, U	melanoma [109]	-
T195C	D-loop	synonymic	not applicable	L0, L2, L3, M, C, E, G, D, N, S3, W, A, X, R, HV, H, J, T, F, B, U, K	melanoma [109]	bipolar disorder [110]
C16261T	D-loop	synonymic	not applicable	L1, M, Q, C, E, N, A, R, P, V, J, T, B, U, K	rectal cancer [101]	-
T16304C	D-loop	synonymic	not applicable	L3, M, I, N, A, R, H, T, U	rectal cancer [101]	-
T6777C	D-loop	synonymic	not applicable	-	epithelial ovarian cancer [108]	-
T16521C	D-loop	synonymic	not applicable	-	stomach cancer [108]	-
G207A	D-loop	synonymic	not applicable	L0, L2, L3, L6, M, Q, C, Z, G, D, N, I, W, A, X, R, H, V, J, T, F, U, K	endometrial cancer [90]	-
C16069T	D-loop	synonymic	not applicable	L0, M, D, N, HV, J	bladder cancer [97]	-
T16189C	D-loop	synonymic	not applicable	M, C, Z, N, S, X, Y, A, F, R, HV1, H, J, T, G, D, L, U, K	endometrial cancer [111], breast cancer, melanoma [109], rectal cancer [112]	coronary artery disease [113] diabetes t.2
T16126C	D-loop	synonymic	not applicable	L0, L1, M, D, N, Y, A, X, JT, R, HV, H, F, B, U	endometrial cancer [90]	-
T16519C	D-loop	synonymic	not applicable	-	breast cancer [69], endometrail cancer [69]	-
C16223A	D-loop	synonymic	not applicable	-	endometrail cancer [90]	-

Abbreviations: LHON—Leber Hereditary Optic Neuropathy, PD—Parkinson’s Disease, ADHD—Attention Deficit Hyperactivity Disorder.
